# Dysregulated transcriptional responses to SARS-CoV-2 in the periphery

**DOI:** 10.1038/s41467-021-21289-y

**Published:** 2021-02-17

**Authors:** Micah T. McClain, Florica J. Constantine, Ricardo Henao, Yiling Liu, Ephraim L. Tsalik, Thomas W. Burke, Julie M. Steinbrink, Elizabeth Petzold, Bradly P. Nicholson, Robert Rolfe, Bryan D. Kraft, Matthew S. Kelly, Daniel R. Saban, Chen Yu, Xiling Shen, Emily M. Ko, Gregory D. Sempowski, Thomas N. Denny, Geoffrey S. Ginsburg, Christopher W. Woods

**Affiliations:** 1 Durham Veterans Affairs Medical Center, Durham, NC USA; 2 Center for Applied Genomics and Precision Medicine, Duke University, Durham, NC USA; 3 Division of Infectious Diseases, Duke University Medical Center, Durham, NC USA; 4 Institute for Medical Research, Durham, NC USA; 5 Duke University Medical Center, Durham, NC USA; 6 Department of Opthalmology, Duke University School of Medicine, Durham, NC USA; 7 Center for Genomics and Computational Biology, Duke University, Durham, NC USA; 8 Duke Human Vaccine Institute, Durham, NC USA

**Keywords:** Viral infection, Infectious-disease diagnostics, SARS-CoV-2

## Abstract

SARS-CoV-2 infection has been shown to trigger a wide spectrum of immune responses and clinical manifestations in human hosts. Here, we sought to elucidate novel aspects of the host response to SARS-CoV-2 infection through RNA sequencing of peripheral blood samples from 46 subjects with COVID-19 and directly comparing them to subjects with seasonal coronavirus, influenza, bacterial pneumonia, and healthy controls. Early SARS-CoV-2 infection triggers a powerful transcriptomic response in peripheral blood with conserved components that are heavily interferon-driven but also marked by indicators of early B-cell activation and antibody production. Interferon responses during SARS-CoV-2 infection demonstrate unique patterns of dysregulated expression compared to other infectious and healthy states. Heterogeneous activation of coagulation and fibrinolytic pathways are present in early COVID-19, as are IL1 and JAK/STAT signaling pathways, which persist into late disease. Classifiers based on differentially expressed genes accurately distinguished SARS-CoV-2 infection from other acute illnesses (auROC 0.95 [95% CI 0.92–0.98]). The transcriptome in peripheral blood reveals both diverse and conserved components of the immune response in COVID-19 and provides for potential biomarker-based approaches to diagnosis.

## Abstract (web summary)

The systemic immune features that distinguish COVID-19 from common infections remain incompletely elucidated. Here McClain et al. compare RNA sequencing in peripheral blood between subjects with SARS-CoV-2 and other respiratory infections and demonstrate dysregulated immune responses in COVID-19 with both heterogeneous and conserved components.

## Introduction

Our understanding of immune mechanisms driving the varied acute, recovery, and post-infectious manifestations of COVID-19 continues to evolve^1^. Recent work has demonstrated altered mRNA profiles in host cells during SARS-CoV-2 infection at the site of infection—in respiratory epithelial cells, BAL, or nasal swab samples—highlighting the dysregulated immune responses at local sites^2–8^. However, the manner in which these signals are modulated (or propagated) beyond the respiratory microenvironment plays a significant role in the ability of the host to control these responses, as suggested by peripheral blood gene expression studies comparing the responses in subjects with COVID-19 to healthy controls^2,9–13^. Specific mechanisms emerging as potential contributors to the pathophysiology driving more severe disease include dysregulation of interferon-stimulated pathways^8,13,14^, modulation of plasmacytoid dendritic cells and NK cell function^12,15^, and hyperactivation of CD8+ T-cell and B-cell compartments^11,15^. However, the manner in which these varied manifestations of SARS-CoV-2 immunity in the periphery differ from those seen in other common respiratory infections is critical to understanding this emerging disease. Transcriptional profiling of peripheral blood samples is an approach that has been shown to elucidate mechanistic underpinnings of inflammatory responses^16^ as well as to identify conserved components of these responses which can offer diagnostic information^17–19^. To further define unique components of the host immune response in subjects with COVID-19, we performed RNA sequencing on whole blood samples from 46 individuals with PCR-positive, symptomatic SARS-CoV-2 infection and compared them directly to subjects with other respiratory infections and healthy controls.

## Results

### Brief overview of study

Subjects with COVID-19 were enrolled when they presented for clinical care, and the time from symptom onset was recorded for each individual sample collected (range 1–35 days). Samples from subjects with COVID-19 were assigned to three groups based on time from symptom onset (early ≤10 days, middle 11–21 days, late >21 days). Fourteen of the SARS-CoV-2 subjects with mild/moderate disease consented to sampling at multiple timepoints, and these were each separated into the appropriate time bin and utilized to control for temporal dynamics of the host response (see Methods). For comparison, we profiled banked blood samples from patients presenting to the emergency department with acute respiratory infection (ARI) due to seasonal coronavirus (*n* = 49), influenza (*n* = 17) or bacterial pneumonia (*n* = 23), and matched healthy controls (*n* = 19). RNA Sequencing was performed on whole blood samples from each relevant subject and timepoint (see Methods for details).

### Transcriptional responses in PBMCs during SARS-CoV-2 infection

**Fig. 1:**
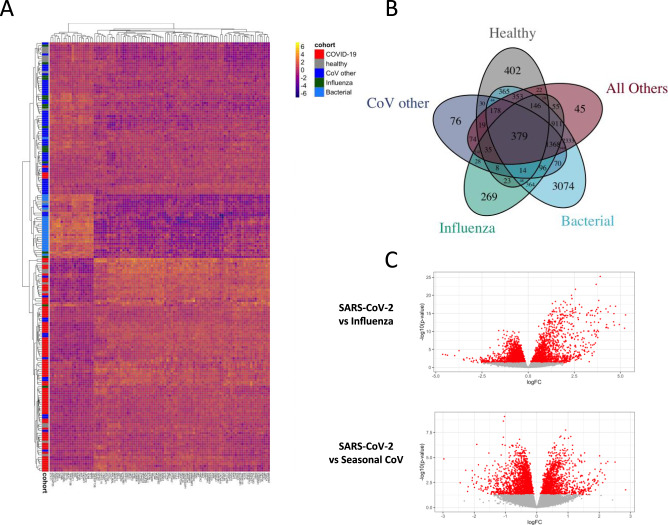
Transcriptomic responses to SARS-CoV-2 in peripheral blood. Heatmap of the top 100 most differentially expressed genes between COVID-19 (*n* = 46) and all other groups (influenza (*n* = 17), seasonal coronavirus (*n* = 49), bacterial pneumonia (*n* = 23), and healthy controls (*n* = 23, **A**)). These represent the 100 genes with the lowest Benjamini–Hochberg adjusted *p* values calculated when comparing COVID-19 to All Others combined. A Venn Diagram demonstrates the number of overlapping genes differentially expressed between COVID-19 subjects and each other infection, healthy controls, or all others combined (**B**, genes shown represent those with adjusted *p* values of < 0.05)). Volcano plot of DEGs in subjects with COVID-19 compared to patients with influenza (**C**, top) and seasonal coronavirus (**C**, bottom).

**Fig. 2:**
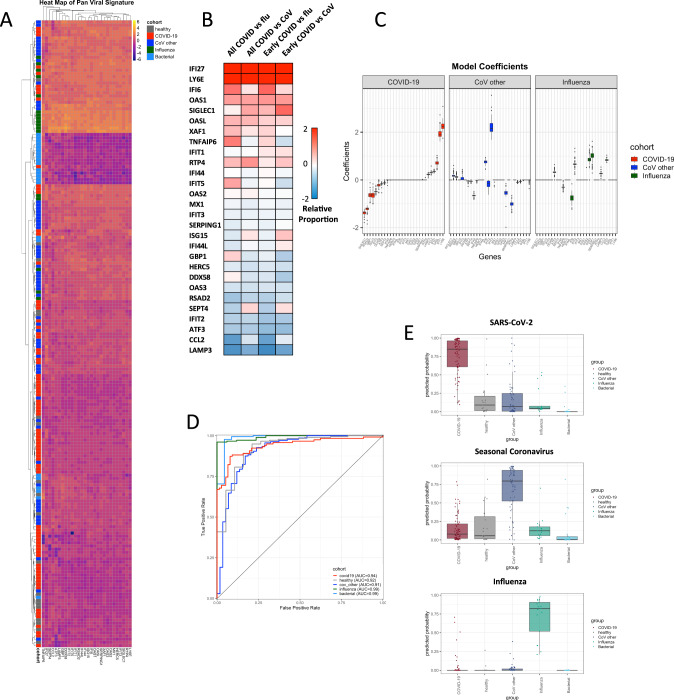
Interferon-related transcriptional signatures. Heatmap of expression of interferon-related genes from a 23-gene signature across all subjects in the study. **A** A number of interferon-stimulated genes are relatively over- or under-expressed in Early (<10 days of symptoms) or all COVID-19 subjects compared to seasonal coronavirus (CoV) or influenza infections (flu, **B**). For comparisons of relative proportions of ISG expression, a logged ratio of per-cohort means was computed for each normalized gene expression value between subjects with COVID-19 and subjects in other groups. Model coefficients (median ± 1.5 times IQR presented, **C**) derived from these relative changes demonstrate the impact of SARS-CoV-2 specific differential ratios of gene expression on overall ISG signature strength (**C**). The 23-gene signature comprised of interferon-stimulated genes discriminates COVID-19 (*n* = 46) from influenza (*n* = 17), seasonal coronavirus (*n* = 49), bacterial pneumonia (*n* = 23), and healthy controls (*n* = 19) across all time points (**D**), while simultaneously identifying seasonal CoV and influenza infections in similar fashion (median probability ± IQR, with whiskers representing 1.5 x IQR, **E**).

Regardless of time from symptom onset, SARS-CoV-2 infection triggered a robust transcriptional response in circulating leukocytes that was markedly different from that seen in other viral or bacterial infections or healthy controls (Fig. 1). At early timepoints (≤10 days of symptoms), the response of most patients was dominated by upregulation of interferon-response signals that have some similarity to those described for other common viral ARIs^17,19–21^ (Fig. 2). Interferon-stimulated genes (ISGs) were generally expressed at a higher level than in healthy subjects but were more muted than seen with seasonal coronaviruses (CoV), and much lower than seen in influenza infection (Figs. 2A and s1, s2). These transcriptional responses were inversely associated with COVID-19 disease duration and viral shedding, declining over time more slowly than is seen with other common viral infections^22–24^. Importantly, these trends were seen within individual subjects as well as across the population as a whole over time (Fig. s2). While many of these ISGs are tightly co-expressed across seasonal CoV and influenza infections, they exhibited bimodal expression in SARS-CoV-2 (Fig. 2B, C). Some ISGs (e.g., *OAS2, IFIT3*) are upregulated similarly to other infectious states while others were dissociated from the common, conserved ISG response and appeared relatively over (*LY6E, OASL, IFI27, IFI6*) or underexpressed (*RSAD2, IFIT2, CCL2, LAMP3*) in SARS-CoV-2 (Fig. 2B). Selective inhibition of Type I IFNs by SARS-CoV and MERS has been well-described^25^, and these observed deviations from what are generally effective interferon responses in seasonal viral infections may contribute to the overall permissive state underlying the prolonged disease course seen with SARS-CoV-2 here and elsewhere^14^.

**Fig. 3:**
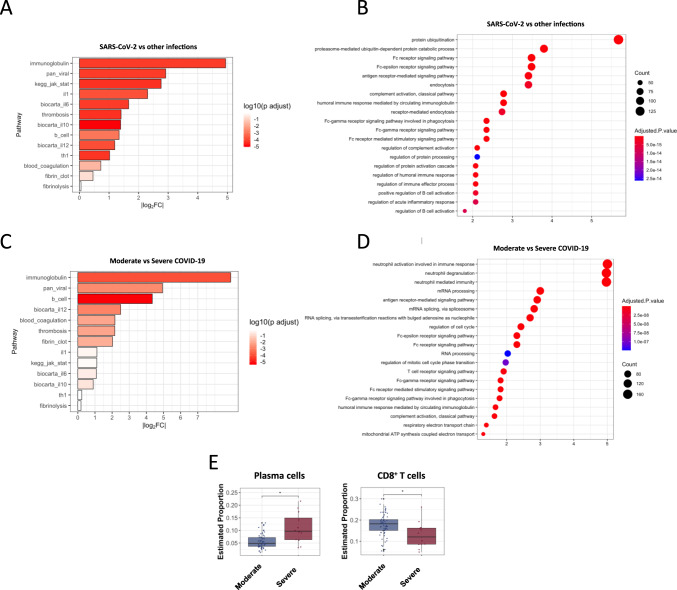
Dysregulated biological pathways in COVID-19. Log_2_FC and significance of changes in relevant curated biological pathways in subjects with COVID-19 (*n* = 46) compared to other infections (*n* = 89, **A**) and in moderate (*n* = 34) vs severe disease (*n* = 12, **C**). Gene Set Enrichment Analysis of the top differentially expressed genes between SARS-CoV-2 (*n* = 46) and other infections (*n* = 89, **B**) and moderate vs severe SARS-CoV-2 (**D**). Relative change in cell type subsets between subjects with moderate and severe COVID-19 was determined through cell type deconvolution of gene expression data, with CIBERSORTx, where linear-mixed models accounting for the multiple-per-subject measurements were used (median proportion ± IQR with whiskers representing 1.5 x IQR is presented,${\,}^*$p = 0.004, Wilcoxon 2-sided signed rank test, **E**).

These data also demonstrate additional transcriptional manifestations of dysregulated biology in COVID-19 subjects relevant to clinical disease. SARS-CoV-2 infection has been associated with clinical and sub-clinical thrombotic events, perhaps due to the hyperinflammatory state^26^, which has led to recommendations for enhanced prophylaxis against venous thromboembolism (VTE) in some hospitalized patients with COVID-19^27^. Compared to other seasonal CoVs and influenza, we observed marked dysregulation of a genomic signature of VTE^28^ (‘thrombosis’, Figs. 3A and s1–s3) and increased expression of many thrombotic pathway genes in a subset of early COVID-19 cases including prekallikrein (*KLKB1*), Factor 12 (*F12*), the plasminogen activator inhibitor (*SERPINE1*) and others, along with decreased expression of antithrombotic protein S (PROS). This signal was most prominent in early disease but persisted in a subset of individuals for as long as 35 days, and was even more prominent in critically ill subjects (Fig. 3C). However, further studies of patients with proven thrombotic or microthrombotic disease will be needed to ascertain whether these changes are directly associated with clinical risk of thrombosis.

We next examined whether inflammatory pathways targeted by putative immunomodulatory therapies demonstrated altered gene expression in these subjects. In a subset of early mild-moderate infections, there was marked dysregulation of IL1, JAK/STAT, IL6, and IL10 signaling pathways compared to other infections (Figs. 3A and  s1, s3). In early COVID-19 there is predominantly muted expression of these pathways, where expression levels more closely resemble healthy controls than seasonal coronavirus or influenza (Fig. s1, s3), which is consistent with permissive hypoinflammatory responses described elsewhere^4–6^. Severely ill subjects exhibited even more marked transcriptional heterogeneity, but showed a trend towards greater IL12 and IFN-response activation along with neutrophil activation, degranulation, and translation initiation, but muted IL1 and IL6 signaling (Fig. 3C, D). They also demonstrate further elevation in plasmablasts/plasma cells compared to mild disease, but decreased proportions of CD8 + T cells (Figs. 3E and s4). While the small number of severely ill subjects (*n* = 12) limits significant conclusion, the heterogeneity seen in expression of target inflammatory pathways across these individuals combined with the suboptimal performance of potential immunomodulatory agents when broadly applied^29,30^, suggests pharmacogenomic approaches to selecting host-directed therapies should be further explored^31^.

**Fig. 4:**
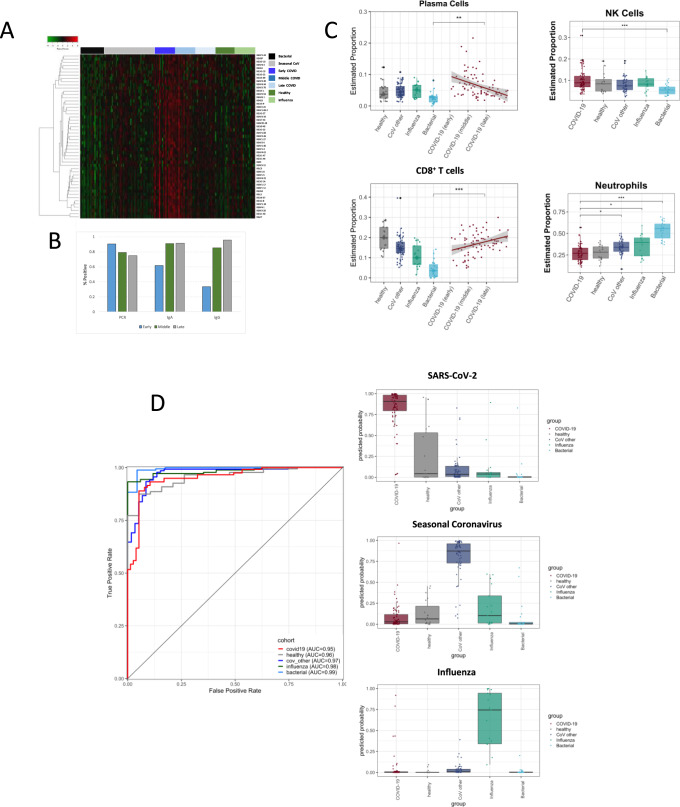
Adaptive immunity and cellular responses to SARS-CoV-2. Indicators of early B-cell activation and immunoglobulin genes are highly upregulated in SARS-CoV-2 compared to other infections (**A** heatmap). Anti-SARS-CoV-2 antibody levels (IgA and IgG) are detectable in a high proportion of subjects with COVID-19 even early in COVID-19 (**B**). These correspond to early elevation of the relative proportion of plasmablasts in SARS-CoV-2 infection (*n* = 77 samples) compare to influenza (*n* = 17), seasonal coronavirus (*n* = 59), bacterial pneumonia (*n* = 23), and healthy controls (*n* = 19, median probability ± 1.5 times IQR presented, **C**). Linear regression was performed to characterize the change of cell-type proportions with respect to time as calculated using CIBERSORTx (**C**, shaded bands represent 95% confidence intervals around trendline, adjusted *P* values: $${\,}^*$$ < 0.05, $${\,}^{**}$$ < 0.001: $${\,}^{***}$$ < 0.0001.). A 139-gene signature, weighted toward immunoglobulin and other genes, similarly discriminates SARS-CoV-2 infected patients (*n* = 46, across all times) from seasonal coronavirus (*n* = 49), influenza (*n* = 17), and bacterial infections (*n* = 23, **D**).

In addition to altered interferon and other inflammatory responses, subjects with early symptomatic COVID-19 exhibited marked upregulation of B-cell activation (*CD79A/B*) and a broad diversity of immunoglobulin genes (*IGHG1, IGHV2-5, IGHV3.30, IGLV3-19, IGLV3-25*, and others) compared to other infectious states (Fig. 4). This distinct transcriptional manifestation of humoral activation occurred as early as 1 day after symptom onset and was highest during the first 7 days before gradually declining throughout the recovery phase of illness (Fig. 4A). This signal corresponded to serum IgA expression as early as the first day of clinical disease, and specific serum IgG expression by day 8 as seen here and elsewhere^32^, and to an early rise in the proportion of plasmablasts/plasma cells relative to other viral infections (Fig. 4B, C). As opposed to the marked heterogeneity seen in inflammatory responses, early B-cell activation was much more tightly conserved, and was magnified further in severely ill subjects (Figs. 3C and s1). Higher antibody titers have been paradoxically associated with disease severity in COVID-19^33,34^ despite evidence for possible therapeutic effect of early administration of exogenous immunoglobulin^35^. It remains to be determined whether these SARS-CoV-2 specific transcriptional findings represent effective early immunity or are part of the virus-induced dysregulation that seems to drive early disease^8,12,15^.

### Discriminatory ability of transcriptomic signatures for COVID-19

Given the strength of the SARS-CoV-2 specific signals, we next explored utilizing linear regression modeling to develop diagnostic signatures for COVID-19. A conserved multivariate transcriptomic signature emerged that differentiated subjects with SARS-CoV-2 infection from all others with a high degree of accuracy (auROC 0.95 [95% CI 0.92–0.98]), regardless of duration of disease or degree of symptoms at the time (Fig. 4D). This 139-gene signature simultaneously identified influenza infection (auROC 0.98 [95% CI 0.96–1]), seasonal coronavirus infections (auROC 0.97 [95% CI 0.95–0.99]), and bacterial pneumonia (auROC 0.99 [95% CI 0.98–1]) without regard for disease severity (Fig. 4D and s5). The SARS-CoV-2 component of this signature was heavily weighted towards Ig-associated genes and transcriptional activation (Table s4). Next, given the uniquely dysregulated interferon response in SARS-CoV-2 infection, we queried the performance of interferon-related gene sets in peripheral blood that have previously been shown to accurately identify viral ARI across a broad array of seasonal viruses^17,21^. One such previously reported “panviral” signature accurately identifies subjects exposed to respiratory viral pathogens prior to symptom onset, often before detectable viral shedding is present^36^. In the current dataset, a 23-gene regression model built from these genes classified the presence or absence of symptomatic SARS-CoV-2 infection with a high degree of accuracy (cross-validated auROC 0.94 [95% CI 0.90–0.97], Fig. 2D). With a change in the relative weights and coefficients of the model, measurement of these 23 mRNAs can also be utilized to diagnose and differentiate COVID-19, seasonal coronavirus, or influenza infections (Fig. 2D, E, and Table s3), consistent with prior work^19^. As with the 139-gene signature, diagnostic accuracy was preserved throughout the prolonged course of COVID-19 in these subjects despite heterogeneity of ISG expression over time, in large part due to the dissociation of some ISGs from one another. Critically, when applied to publicly available data from a separate cohort^9^, both the 23-gene and 139-gene signatures also accurately differentiated SARS-CoV-2 infection from healthy controls (*p* < 0.001, Fig. s6).

## Discussion

Analysis of transcriptional responses in peripheral blood from patients with SARS-CoV-2 infection reveals that SARS-CoV-2 triggers inflammatory and humoral immune response pathways in ways that are distinct from those seen in other common respiratory infections. These SARS-CoV-2 specific signals further support the growing body of evidence that dysregulated immunity likely contributes to early viral shedding^8^ and progression to severe disease^9,37,38^. The marked heterogeneity seen in many inflammatory pathways underscores the potential importance of defining the pathophysiology of infection at the individual level, especially when considering immunomodulatory therapy^31^. However, despite observed heterogeneity in many immune responses, these data show that some components of the host transcriptional response to SARS-CoV-2 infection are highly conserved. The potential for a diagnostic approach based on these findings is profound, as they raise the possibility that measurement of expression levels of a small set of genes could detect SARS-CoV-2 infection as well as simultaneously providing information about the presence of other viral (or even bacterial) infections. Also, since signatures built on similar genes have been shown to accurately detect early, even pre-symptomatic influenza and seasonal coronavirus infections, it is possible that tests measuring these signatures may similarly detect COVID-19 in exposed individuals^9^. When combined with emerging nucleic acid detection platforms that offer sample-to-answer times measured in minutes, successful demonstration of pre-symptomatic COVID-19 detection could contribute to real-time outbreak surveillance and quarantine decisions for asymptomatic but potentially contagious hosts that drive much of the spread of this disease^39^. These results further support the growing body of literature demonstrating the efficacy of measurement of the host transcriptome as an adjunct diagnostic approach for respiratory infections^17,36,40,41^, although they will clearly require additional validation in larger cohorts of subjects with COVID-19 as they become available. Given the wide array of existing and emerging RT-PCR-based platforms capable of measuring host gene expression, the discovery of multiple high-performing transcriptomic signatures in peripheral blood based on different aspects of the unique host response to SARS-CoV-2 reinforces the promise of developing stable, reproducible, and flexible point-of-care host response assays to aid in detection and control of COVID-19.

Together with prior studies^4,6,9^, these data demonstrate that the transcriptional landscape of the host response to SARS-CoV-2 infection is robust, can elucidate key biological mechanisms of disease, may prove useful for therapeutic drug selection, and contains conserved components which show promise for a new generation of host-based diagnostics to combat this devastating disease.

## Methods

### Institutional review board approvals

The relevant protocols were approved by the IRBs of participating institutions, and were conducted in accordance with the Declaration of Helsinki, applicable regulations and local policies.

### Clinical cohort enrollment

Patients with confirmed SARS-CoV-2 infection were identified through the Duke University Health System (DUHS) or the Durham Veterans Affairs Health System (DVAHS) and enrolled into the Molecular and Epidemiological Study of Suspected Infection (MESSI, Pro00100241) with clinical data compiled into REDCap. RT-PCR testing for SARS-CoV-2 was performed at either the North Carolina State Laboratory of Public Health or through the clinical laboratory at either DUHS or DVAHS. Subjects with COVID-19 were divided into early, middle, and late disease based on time from reported symptom onset (early ≤10 days, middle 11–21 days, late >21 days), and were divided into severity categories based on degree of illness (“mild/moderate” for outpatients and “severe” for subjects requiring hospitalization). Fourteen subjects with SARS-CoV-2 infection (all outpatients with mild/moderate disease) also consented to sampling at multiple time points (day of enrollment, day 7 and day 14 from enrollment). Subjects with acute respiratory illness of alternative etiologies including seasonal coronavirus, influenza, or bacterial etiologies were prospectively enrolled from a Duke University undergraduate cohort (Predicting Health and Disease, Pro00082317); emergency departments at DUHS, DVAHS, Henry Ford Hospital, or University of North Carolina as part of the CAPSOD (Community-Acquired Pneumonia and Sepsis Outcome Diagnostics, ClinicalTrials.gov NCT00258869), CAPSS (Community-Acquired Pneumonia and Sepsis Study), or RADICAL (Rapid Diagnostics in Categorizing Acute Lung Infection) studies (Tables s1, s2). Written informed consent was obtained from all subjects or legally authorized representatives. All subjects enrolled in CAPSOD, CAPSS, and RADICAL underwent clinical adjudication to determine the etiology of infection^19^. Multiplex viral PCR testing was performed for all subjects using the ResPlex 2•0 viral PCR multiplex assay (Qiagen), xTAG RVP FAST 2 (Luminex), or NxTAG Respiratory Pathogen Panel (Luminex).

### RNA sequencing

Peripheral blood was collected in PAXgene™ Blood RNA tubes (PreAnalytiX), and total RNA extracted using the PAXgene™ Blood miRNA Kit (Qiagen) employing the manufacturer’s recommended protocol. RNA quantity and quality were assessed using Nanodrop 2000 spectrophotometer (Thermo-Fisher) and Bioanalyzer 2100 with RNA 6000 Nano Chips (Agilent). RNA sequencing libraries were generated using NuGEN Universal mRNA-seq kit with AnyDeplete Globin (NuGEN Technologies, Redwood City, CA) and sequenced on the Illumina NovaSeq 6000 instrument with an S4 flow cell (50 million paired-end read clusters per sample with 100 bp read length; performed through the Duke Sequencing and Genomic Technologies Core). Antibody response testing was performed using the anti-SARS-CoV-2 IgG ELISA assay (EUROIMMUN Medizinische Labordiagnostika AG, Lübeck, Germany) according to the manufacturer’s instructions. Test results were evaluated by calculating the ratio of the OD (optical density) of the test sample over the OD of the calibrator sample. Ratio of <0.8 was interpreted as negative and ratio of 1.1 or greater as positive (ratio of 0.8 to <1.1 as indeterminate and not utilized in phenotyping).

### Statistical analysis

RNA Sequencing data was normalized using the frozen RMA method^42^. The sequencing reads were trimmed and aligned to the human reference genome GRCh38 and a count matrix obtained utilizing STAR v2.7.1a^43^. Genes with counts per million greater than 1 in fewer than 20% of samples were dropped along with three samples with a high proportion of lowly expressed reads. The data was normalized using trimmed mean normalization^44^ and then log2 transformed.

We first performed univariate testing between the COVID-19 subjects and all others (healthy, Influenza, CoV other, Bacterial), both as COVID-19 against a single other group and as COVID-19 against all other groups at once. Additionally, we repeated these analyses with the COVID-19 subjects divided into early, middle, and late disease by time since symptom onset. Generalized linear models for univariate testing were implemented using the limma package in R^45^ and this was utilized to account for correlations between multiple measurements from the same individual over time. For each comparison, we report multiple hypothesis testing corrected *p* values (Benjamini–Hochberg).

Next, we identified differentially expressed pathways between the groups of interest by repeating the above comparisons and performing a similar univariate testing procedure. Gene pathway and upstream regulator analysis was performed with EnrichR. The normalized expression of the genes in each pathway was summarized as their first principal component (PC). These PCs were then used for univariate testing. We computed coordinates of our samples with respect to the first PC to obtain a dataset of pathway “expressions”, exactly analogous to the gene expressions previously tested.

Finally, we trained a statistical model that predicts the group label that a subject belongs to. We fit a sparse multinomial logistic regression model to the data^46^. We performed parameter selection and performance estimation via a nested leave-one-out cross validation procedure on the subjects. We used the glmnet package in R^46^ for the basis of our implementation. Performance was estimated in terms of area under the curve (AUC) of the receiving operating characteristic (ROC) for binary comparisons involving COVID-19 vs other groups.

### Validation cohort

We further evaluated performance of the two primary gene expression signatures using a publicly available peripheral blood single cell RNA (scRNA) sequencing dataset^9^ containing eight samples from subjects with COVID-19 and six healthy age-matched controls (NCBI Gene Expression Omnibus #GSE150728). We pre-processed droplet-based scRNA data (count matrices were built from the BAM files using dropEst 0.8.6) and filtered out low quality cells and genes (cells that had fewer than 1000 UMIs or greater than 15,000 UMIs, as well as cells that contained greater than 20% of reads from mitochondrial genes or rRNA genes were considered low quality and removed from further analysis). A gene by sample matrix was generated by summing raw expression of the cells (without scaling and transformation) from each sample. Expression of the genes whose median coefficient values (from the model) are non-zero for COVID-19 in both the 139-gene signature and the 23-gene signature were compared across clinical phenotypes in the validation dataset using the Mann–Whitney U test.

### Analysis of estimated cell type proportions

The CIBERSORTx method was used to estimate cell-type proportions and perform batch correction for platform differences^47^. A validated signature matrix (LM22) derived from microarray data with 22 human hematopoietic populations was used. Estimated cell types were grouped into primary categories. A linear-mixed model was used to test for differences in etiologies, time from symptom onset, and hospital-admission status. The model accounted for the multiple-per-subject measurements. *P* values were adjusted for multiple comparisons using the Benjamini–Hochberg method. Additionally, appropriate samples were available for a small subset of SARS-CoV-2 subjects (*n* = 12) for flow cytometric analysis for comparison to the calculated COVID-19-associated changes in cell type proportions from RNA Sequencing data (Fig. s7, Supplemental Methods).

### Reporting Summary

Further information on research design is available in the Nature Research Reporting Summary linked to this article.

## Supplementary information

Supplementary Information

Reporting Summary

## Data Availability

The RNA Sequencing datasets generated during and/or analyzed during the current study are publicly available through the National Center for Biotechnology Information Gene Expression Omnibus, accession# GSE161731.
