# Supplementary material for: Dysregulated transcriptional responses to SARS-CoV-2 in the periphery
Source: Nat Commun. 2021 Feb 17;12:1079. doi: 10.1038/s41467-021-21289-y (PMC7889643; doi:10.1038/s41467-021-21289-y)

Supplementary Material

Page:

1 **List of Online Materials**

2 **Table s1** Demographics

3 **Table s2** Clinical data on Hospitalized Subjects

4 **Table s3** Genes and coefficients in the multivariate 23-gene ISG classification model

5 **Table s4** Genes and coefficients in the multivariate 139-gene model for diagnosis of COVID-19.

9 **Figure s1** Dysregulated biological pathways in COVID-19.

10 **Figure s2** Comparison of population-based and within-host dynamics of interferon-stimulated gene expression over time.

11 **Figure s3** Biological dysregulation over time in COVID-19

12 **Figure s4:** Proportional cell type breakdown across clinical phenotypes and time

13 **Figure s5** Model performance across disease severity.

14 **Figure s6** Performance of discriminatory models in a published cohort of patients with SARS-CoV-2 infection and Healthy controls*.

15 **Figure s7** Proportional cell type breakdown across clinical phenotypes and time

16 **Supplement Methods**

Table s1: Demographics of study population

	COVID-19	Seasonal CoV	Influenza	Bacterial	Healthy
**Gender:**					
Male	24 (52%)	23 (45%)	8 (47%)	10 (32%)	11 (58%)
Female	22 (48%)	28 (55%)	9 (53%)	13 (57%)	8 (42%)
**Age** (range):					
	46 (20-91)	21 (18-59)	23 (14-51)	62 (33-87)	18 (18-20)
**Race**, n (%)					
Native American	1 (2%)	2 (4%)	1 (6%)	0	0
Asian	6 (13%)	10 (20%)	0	0	7 (37%)
African American	10 (21%)	6 (12%)	5 (29%)	19 (83%)	1 (5%)
White	28 (61%)	32 (64%)	10 (59%)	4 (17%)	11 (58%)
Other	1 (2%)	1 (2%)	1 (6%)	0	0

	1	2	3	4	5	6	7	8	9	10	11	12
**Age (years)**	60	91	64	76	69	33	88	69	59	71	31	67
**Sex**	Male	Female	Male	Male	Male	Male	Female	Female	Male	Male	Female	Male
**Race**	Black	Black	White	White	Black	White	Black	Black	Black	Black	Black	Black
**Ethnicity**	Non-hispanic	Non-hispanic	Non-hispanic	Non-hispanic	Non-hispanic	Non-hispanic	Non-hispanic	Non-hispanic	Non-hispanic	Non-hispanic	Unknown	Non-hispanic
**Chronic medical conditions**	CKD	COPD, CAD, HTN obesity, sleep apnea	aortic aneurysm, CAD, CVA, HTN, obesity, prior tobacco use	Asthma, CAD, diabetes, HTN, prostate cancer	CAD, CKD, CVA, diabetes, HTN	None	CAD, CVA, HTN, obesity, pulmonary emboli	CVA, HTN, obesity, reactive airways, SLE	diabetes, HTN, obesity, sleep apnea	HTN, multiple sclerosis, post-traumatic stress disorder, tobacco use	Pregnancy, sleep apnea, prior tobacco use	Diabetes, heart failure, HTN, obesity, paraplegia, vascular disease
**Days of symptoms prior to enrollment**	13	7	14	3	2	9	14	5	16	1	18	11
**O2 at enrollment**	0-1L	1-2L	0L	0-2L	1-3L	0-1L	0L	0L	1L	6L	50% FiO2	45% FiO2
**Initial therapy**	None	Remdesivir	None	None	HCQ/azithromycin	HCQ	None	None	Remdesivir	None	HCQ, remdesivir, tocilizumab	None
**Days on therapy prior to enrollment**	--	1	--	--	1	0	--	--	3	--	9	--
**Duration of hospitalization (days)**	3	11	3	7	3	1	21	3	8	13	43	42
**ICU**	No	No	No	No	No	No	No	No	Yes	Yes	Yes	Yes
**Mechanical Ventilation**	No	No	No	No	No	No	No	No	No	No	Yes	Yes
**Deceased**	No	Yes	No	Yes	Yes	No	No	No	No	No	No	No

CAD: coronary artery diseases
CKD: chronic kidney disease
COPD: chronic obstructive pulmonary disease
CVA: cerebrovascular accident
HTN: hypertension
SLE: systemic lupus erythematosus
HCQ: hydroxychloroquine

Table s3. Genes and coefficients in the multivariate 23-gene ISG classification model

	COVID-19	Healthy	Seasonal CoV	Influenza	Bacterial
**LY6E**	2.238	0.000	0.000	0.000	0.000
**IFIT1**	1.891	1.412	0.000	0.000	-0.312
**SIGLEC1**	-1.364	0.388	0.000	0.000	0.000
**RSAD2**	-1.205	0.000	0.172	0.000	0.000
**OASL**	0.710	0.000	0.000	0.000	0.000
**GBP1**	-0.631	0.000	0.127	0.000	0.000
**ISG15**	-0.629	0.584	0.000	0.000	0.000
**IFIT5**	-0.492	0.000	0.009	0.000	0.000
**IFI27**	0.349	-0.329	-0.058	0.829	0.000
**CCL2**	0.305	0.000	-0.099	0.000	0.102
**LAMP3**	0.230	-0.122	0.000	0.269	-0.296
**DDX58**	-0.215	0.000	0.000	0.000	0.000
**ATF3**	-0.163	0.000	-0.046	0.325	0.000
**SEPT4**	-0.124	0.000	-0.061	0.000	0.000
**IFI6**	0.000	0.000	0.760	-0.749	0.000
**IFI44**	0.000	1.030	-0.655	0.000	0.000
**TNFAIP6**	0.000	0.465	-0.013	-0.308	2.257
**RTP4**	0.000	0.186	-1.001	0.000	-1.102
**SERPING1**	0.000	-0.524	0.000	0.000	0.000
**IFIT2**	0.000	0.000	-0.157	0.658	0.000
**IFIT3**	0.000	0.000	2.156	0.000	0.000
**OAS3**	0.000	-3.156	0.000	0.844	0.000
**XAF1**	0.000	0.000	-0.536	1.009	0.000

Table s4. Genes included in the multivariate model for diagnosis of COVID-19.

	COVID-19	CoV other	Influenza	Bacterial	Healthy
**AKT2**	1.1230	0	0	0	0
**LPCAT1**	1.0216	0	0	0	0
**CACNA1I**	0.7649	0	0	0	0
**PTMAP5**	0.6749	0	0	0	0
**TMEM184B**	0.5090	0	0	0	0
**C15ORF52**	0.3640	0	0	0	0
**PIF1**	0.2581	0	0	0	0
**RPL36AL**	0.2106	0	0	0	0
**RHOB**	0.2022	0	0	0	0
**KLHDC8B**	0.1923	0	0	0	0
**CETN2**	0.1825	0	0	0	0
**IGHV1.24**	0.1341	0	0	0	0
**KEAP1**	0.1195	0	0	0	0
**PDHB**	0.1081	0	0	0	0
**HNRNPA1P21**	0.0659	0	0	0	0
**CD70**	0.0643	0	0	0	0
**BIRC5**	0.0572	0	0	0	0
**NKX3.1**	0.0510	0	0	0	0
**HOXA.AS2**	0.0459	0	0	0	0
**TBC1D19**	0.0441	0	0	0	0
**IGHG1**	0.0365	0	0	0	0
**PTRF**	0.0354	0	0	0	0
**IGKV1.9**	0.0287	0	0	0	0
**WASH5P**	0.0222	0	0	0	0
**IGHGP**	0.0163	0	0	0	0
**RP11.81H14.2**	0.0054	0	0	0	0
**RP11.707O23.5**	0.0044	0	0	0	0
**PM20D1**	0.0018	0	0	0	0
**TREML4**	-0.0005	0	0	0	0
**KIAA2018**	-0.0038	0	0	0	0
**RP11.861A13.3**	-0.0075	0	0	0	0
**AC092299.1**	-0.0083	0	0	0	0
**NUTM2B.AS1**	-0.0125	0	0	0	0
**NRG1**	-0.0152	0.0952	0	0	0
**RP13.270P17.3**	-0.0158	0	0	0	0
**ZNF607**	-0.0227	0	0	0	0
**PTGES3P1**	-0.0326	0	0	0	0
**RP11.180M15.7**	-0.0383	0	0	0	0
**ZFHX3**	-0.0496	0	0	0	0

**RP11.358B23.7**	-0.0583	0	0	0	0
**CTD.2357A8.2**	-0.0709	0	0	0	0
**PRRG4**	-0.0747	0	0	0	0
**MRAS**	-0.0794	0	0	0	0
**HORMAD1**	-0.1087	0	0	0	0
**CTD.2047H16.3**	-0.1134	0	0	0	0
**MDGA1**	-0.1496	0	0	0	0
**PCGF3**	-0.1539	0	0	0	0
**FAM13A.AS1**	-0.1594	0	0	0	0
**RP11.925D8.3**	-0.1836	0	0	0	0
**AOAH.IT1**	-0.1955	0	0	0	0
**NID1**	-0.2495	0	0	0	0
**TRAF6**	-0.5686	0	0	0	0
**HEATR5B**	-0.7364	0	0	0	0
**TNFSF12**	0	0.5862	0	0	0
**HLA.B**	0	0.5808	0	0	0
**FLVCR1**	0	0.5327	0	0	0
**IL34**	0	0.2339	0	0	0
**ALAS2**	0	0.2290	0	0	0
**IFI6**	0	0.1947	0	0	0
**CPVL**	0	0.1503	0	0	0
**AP1S2**	0	0.1385	0	0	0
**C21ORF33**	0	0.1124	0	0	0
**HRK**	0	0.1060	0	0	0
**RP11.363E7.4**	0	0.0705	0	0	0
**FAM124B**	0	0.0669	0	0	0
**NONO**	0	0.0611	0	0	0
**NRCAM**	0	0.0591	0	0	0
**MYCL**	0	0.0574	0	0	0
**CD1E**	0	0.0434	0	0	0
**PDE7B**	0	0.0429	0	0	0
**TRDV2**	0	0.0408	0	0	0
**RP1.197B17.4**	0	0.0341	0	0	0
**MT.CO3**	0	0.0258	0	0	0
**ARAP1.AS2**	0	0.0105	0	0	0
**IL20RB**	0	0.0071	0	0	0
**SERPINB9**	0	0.0032	0	0	0
**XIST**	0	0.0007	0	0	0
**SIDT2**	0	0	1.3428	0	0
**TRIB2**	0	0	0.6096	0	0
**CDKN1A**	0	0	0.5817	0	0
**GNPDA1**	0	0	0.2763	0	0
**KIAA0226**	0	0	0.2753	0	0

**IFI27**	0	0	0.0988	0	-0.0508
**CXCR2P1**	0	0	0.0982	0	0.0000
**H1F0**	0	0	0.0087	0	-0.5679
**LTA4H**	0	0	0	0.5692	0
**VAPB**	0	0	0	0.5088	0
**PRPF38B**	0	0	0	0.4198	0
**FAM105A**	0	0	0	0.3718	0
**MAFG**	0	0	0	0.3365	0
**FBXW2**	0	0	0	0.3113	0
**TMCO3**	0	0	0	0.0314	0
**RETN**	0	0	0	0.0297	0
**ZNF3**	0	0	0	0	1.2052
**PMPCB**	0	0	0	0	1.0779
**RPL6**	0	0	0	0	0.9235
**RPS24**	0	0	0	0	0.4027
**TMA7**	0	0	0	0	0.3479
**RP11.244J10.1**	0	0	0	0	0.3075
**EEF1B2**	0	0	0	0	0.0120
**RIMBP3**	0	0	0	0	-0.0205
**PWP2**	0	0	0	0	-0.0520
**TROAP**	0	0	0	0	-0.0817
**DPRXP2**	0	0	0	0	-0.1304
**CASC5**	0	0	0	0	-0.2287
**PTGES3**	0	0	0	0	-0.8241
**CACNG6**	0	0	0	-0.0028	0
**RNASEH2B**	0	0	0	-0.0929	0
**TRBV4.2**	0	0	0	-0.2042	0
**FXN**	0	0	0	-0.2882	0
**FLT4**	0	0	-0.0109	0	0
**KIAA1045**	0	0	-0.0219	0	0
**LINC00998**	0	0	-0.0444	0	0
**THBD**	0	0	-0.1061	0	0
**RAPGEFL1**	0	0	-0.1466	0	0
**CD302**	0	0	-0.3018	0	0
**PI3**	0	0	-0.3203	0	0
**LRP3**	0	0	-0.4588	0	0
**RP11.696N14.1**	0	-0.0042	0	0	0
**CHRNA2**	0	-0.0046	0	0	0
**COX17**	0	-0.0133	0	0	0
**HIST1H2BO**	0	-0.0152	0	0	0
**AAGAB**	0	-0.0305	0	0	0
**RP1.34B20.4**	0	-0.0809	0	0	0
**RPL37AP1**	0	-0.0810	0	0	0

**CYP2J2**	0	-0.0997	0.0046	0	0
**C19ORF84**	0	-0.1228	0	0	0
**HMGB1P5**	0	-0.1449	0	0	0
**PSMD5.AS1**	0	-0.1498	0	0	0
**HIST1H2BH**	0	-0.1669	0	0	0
**HIST1H2BF**	0	-0.1897	0	0	0
**WDR55**	0	-0.2295	0	0	0
**SMG5**	0	-0.3437	0	0	0
**CWC15**	0	-0.3828	0	0	0
**MSC**	0	-0.3837	0	0	0
**LUC7L3**	0	-0.4616	0	0	0
**GOLGA5**	0	-0.4821	0	0	0
**SNW1**	0	-0.5709	0	0	0
**BSDC1**	0	-1.2532	0	0	0

Figure s1: Dysregulated biological pathways in COVID-19. Log_2FC for each gene in each pathway across relevant groups is represented by a point. Median log_2FC +/- IQR, with whiskers representing 1.5 x IQR for all genes in the pathway are depicted. Comparisons represent Early COVID-19 (0-10 days from symptom onset, n=19 samples, A), Middle COVID-19 (11-21 days, n=36 samples, B), and Late COVID-19 (>21 days since symptom onset, n=22 samples, C) are each compared to Healthy controls (n=23 samples). Early COVID-19 patients are further divided into outpatients (Moderate, n=34 subjects) and inpatients (Severe, n=12 subjects, D).

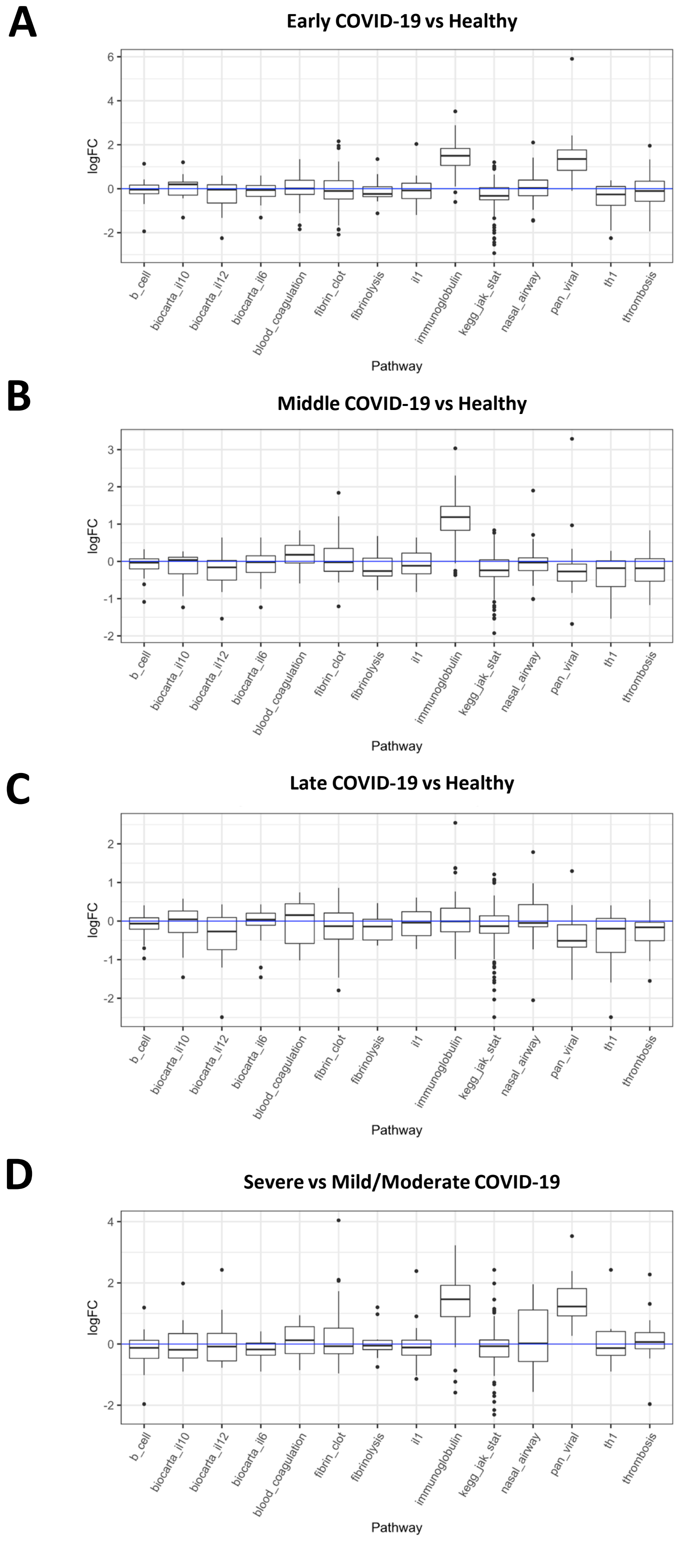

Figure s2: Comparison of population-based and within-host dynamics of interferon-stimulated gene expression over time. Normalized expression of top ISGs in patients with COVID-19 (n=46) compared to influenza (n=17), seasonal coronavirus (n-49), bacterial pneumonia (n=23) and healthy controls (n=23) are presented. Additionally, 14 of the 46 subjects with COVID-19 had serial timepoints collected (enrollment, day 7, and day 14 of study). Duration of symptoms at the time of enrollment for these subjects varied from 5 to 21 days. For each of the top 8 interferon-stimulated genes (as determined by strength of coefficients contributing to the 23-gene signature), expression levels across cohorts (by etiology) are presented (boxplots, median +/- IQR, with whiskers representing 1.5 x IQR), followed by granular presentation of expression level of the gene in each COVID-19 subject at Early (n=19 samples), Middle (n=36 samples) and Late times (n=22 samples, red dots). Red lines connect samples from an individual COVID-19 subject across time, and shaded bands represent 95% confidence interval around the trendline..

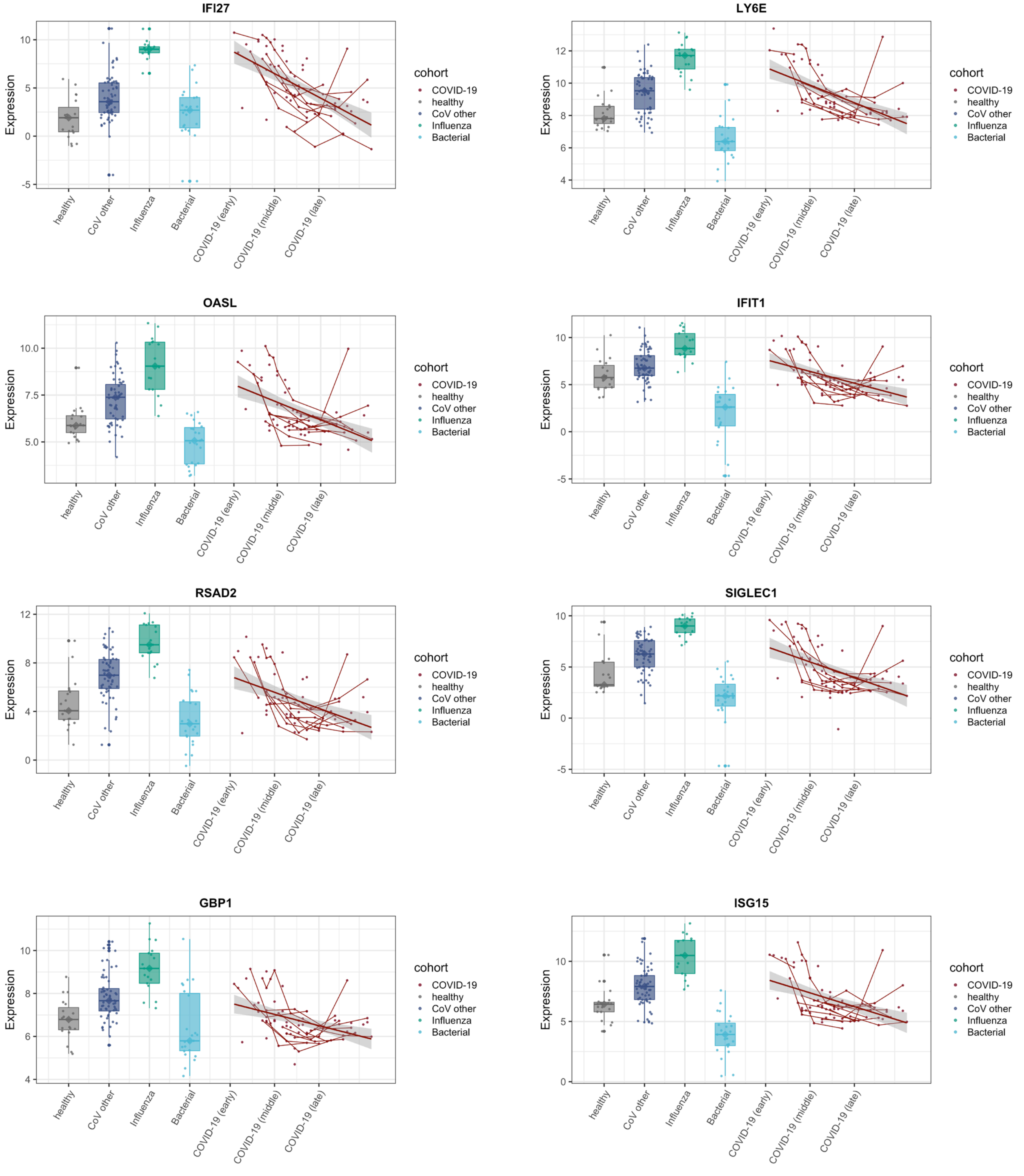

Figure s3: Biological dysregulation over time in COVID-19. Magnitude and variance associated with the first principle component of each biological pathway across time in subjects with COVID-19 (n=46), influenza (n=17), seasonal coronavirus (n=49), bacterial pneumonia (n-23), and healthy controls (n=19) . Marked heterogeneity is seen across patients with COVID-19. A subset of SARS-CoV-2 infected subjects exhibit activation/suppression of these pathways compared to healthy subjects, most notably at early times, although for some subjects these alterations persist for weeks.

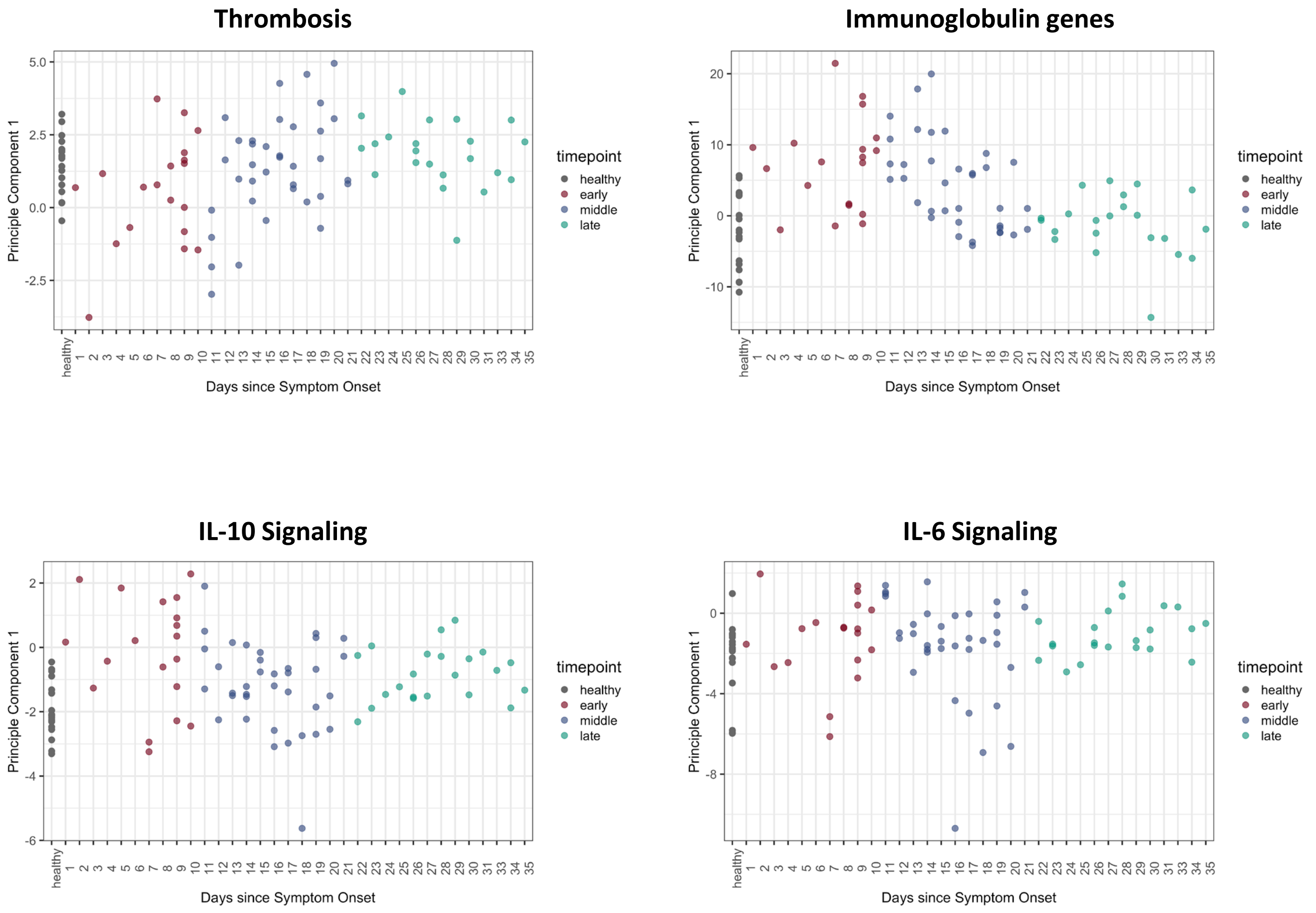

Figure s4: Proportional cell type breakdown across clinical phenotypes and time. The CIBERSORTx method was used to estimate cell-type proportions in patients with COVID-19 (n=46) compared to influenza (n=17), seasonal coronavirus (n-49), bacterial pneumonia (n=23) and healthy controls (n=23). Relative proportions of each cell type in each subjects from each clinical category as determined using CIBERSORTx are presented. Subjects with COVID-19 are further subdivided by time from symptom onset (Early <10 days, Middle 11-20 days, Late >20 days). Median +/- IQR, with whiskers representing 1.5 x IQR is depicted. Linear regression was performed to characterize the change of cell type proportions with respect to time (red lines). Shaded bands represent 95% confidence intervals around the trendline.*adjusted P-value < 0.05, **adjusted P-value < 0.001: *** adjusted P-value < 0.0001.

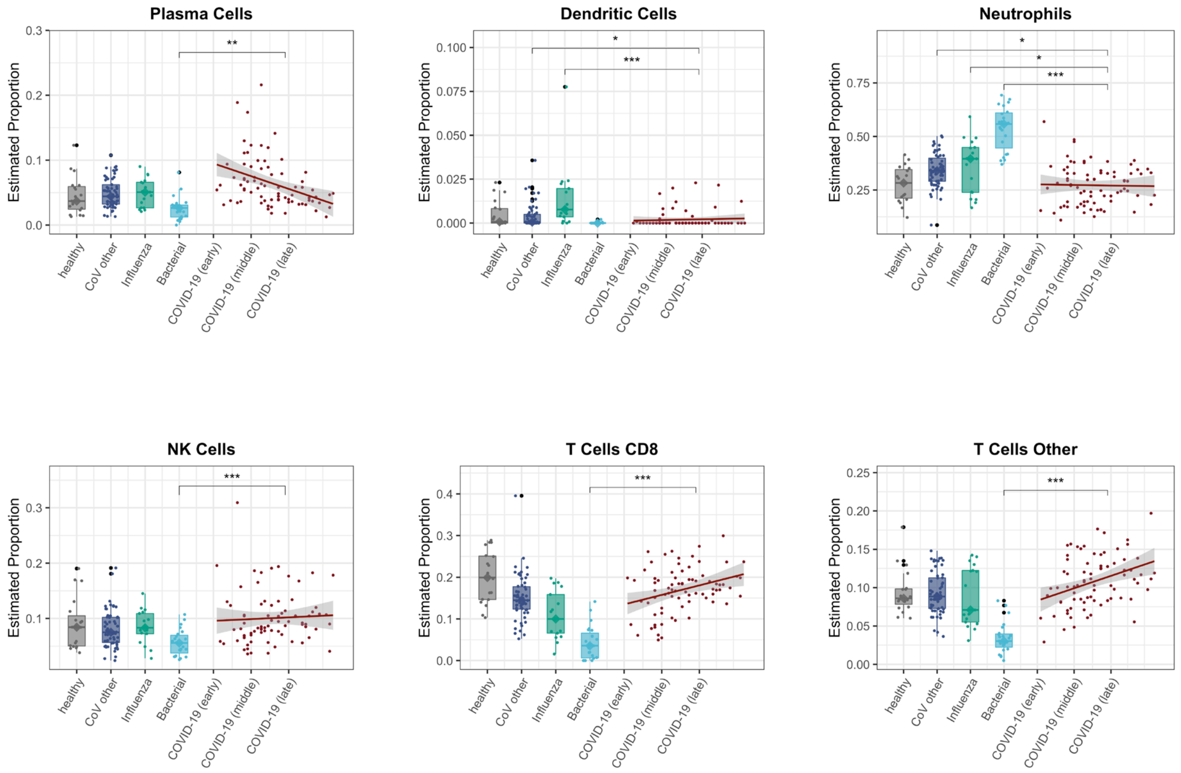

Figure s5: Model performance across disease severity. Performance of discriminatory models is not significantly different between outpatient (A,C) and hospitalized (B,D) subjects for either the 23-gene model (A,B), or for the 139-gene model (C,D).

A

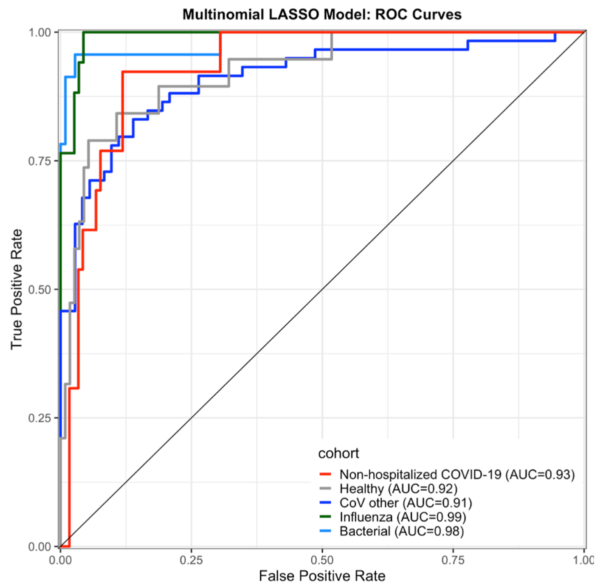

B

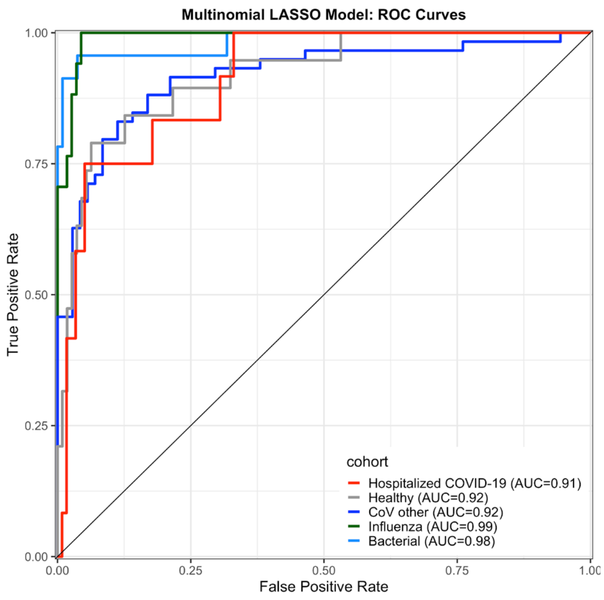

C

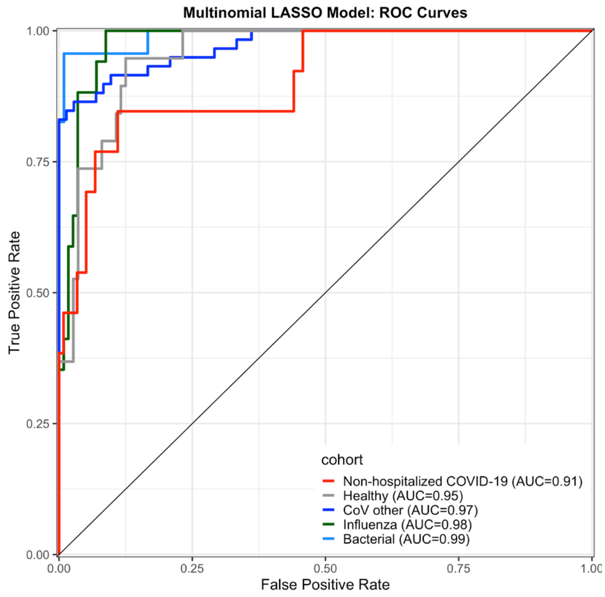

D

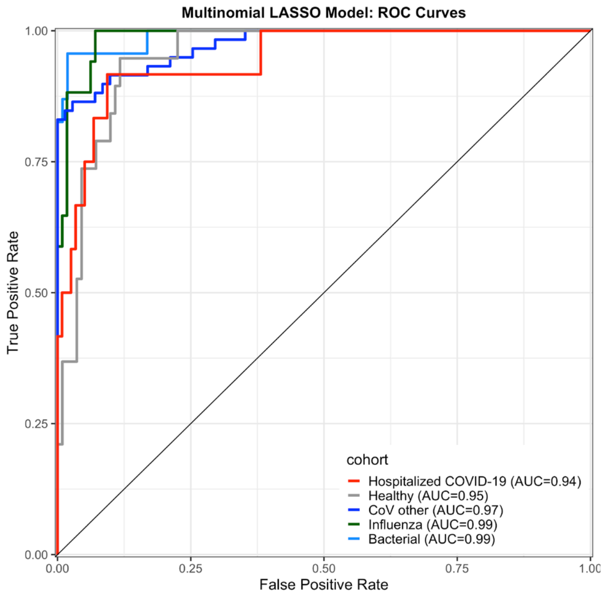

Figure s6: Performance of discriminatory models. Expanded performance metrics of both models in the discovery cohort (Table) and in a separate published cohort of patients with SARS-CoV-2 infection (n=8) and Healthy controls (n=6)*.

	ACC*	TPR	FPR	PPV	NPV
**23-gene**	0.89 (0.85-0.93)	0.92 (0.85-0.97)	0.13 (0.06-0.18)	0.83 (0.75-0.91)	0.94 (0.90-0.98)
**139-gene**	0.91 (0.89-0.96)	0.95 (0.86-0.99)	0.11 (0.03-0.15)	0.85 (0.80-0.95)	0.96 (0.91-0.99)

* Accuracy (ACC), True Positive Rate (TPR), False Positive Rate (FPR), Positive Predictive Value (PPV), Negative Predictive Value (NPV). All values calculated at point of optimal Youden Index.

Performance of 139-gene model in Wilk et al dataset

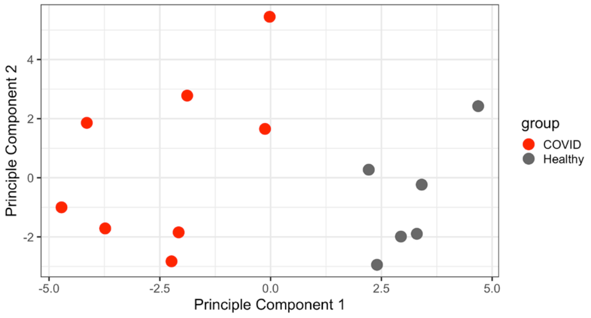

139-gene model†

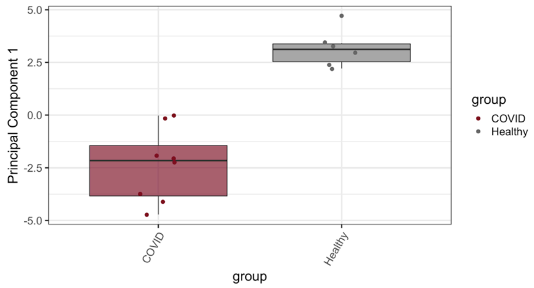

Performance of 23-gene PanViral Model genes in Wilk et al dataset

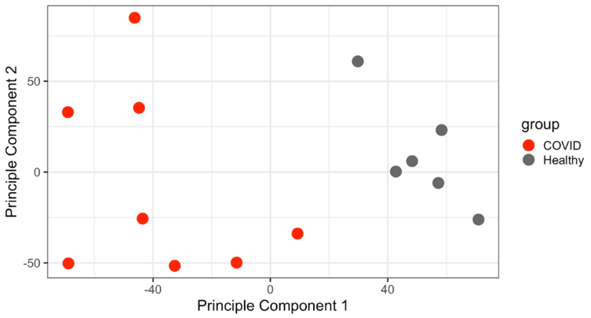

23-gene model†

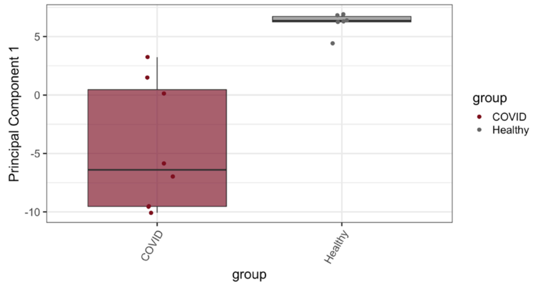

*Wilk, A.J., et al. A single-cell atlas of the peripheral immune response in patients with severe COVID-19. Nature medicine (2020).
† p<0.001, median +/- IQR, with whiskers representing 1.5 x IQR is depicted

Figure s7: Cell type deconvolution of gene expression data compared to flow cytometric measurement of cell proportions. Correlation between the two measurements for cell types which are significantly during SARS-CoV-2 infection (n=12 subjects) are presented (top) as are direct comparisons of the estimated and measured proportions of cells across the two methodologies (bottom, median $\log_2FC$ +/- IQR, with whiskers representing 1.5 x IQR). Linear regression was performed to characterize the change of cell-type proportions with respect to time. Shaded bands represent 95% confidence intervals around the trendline.

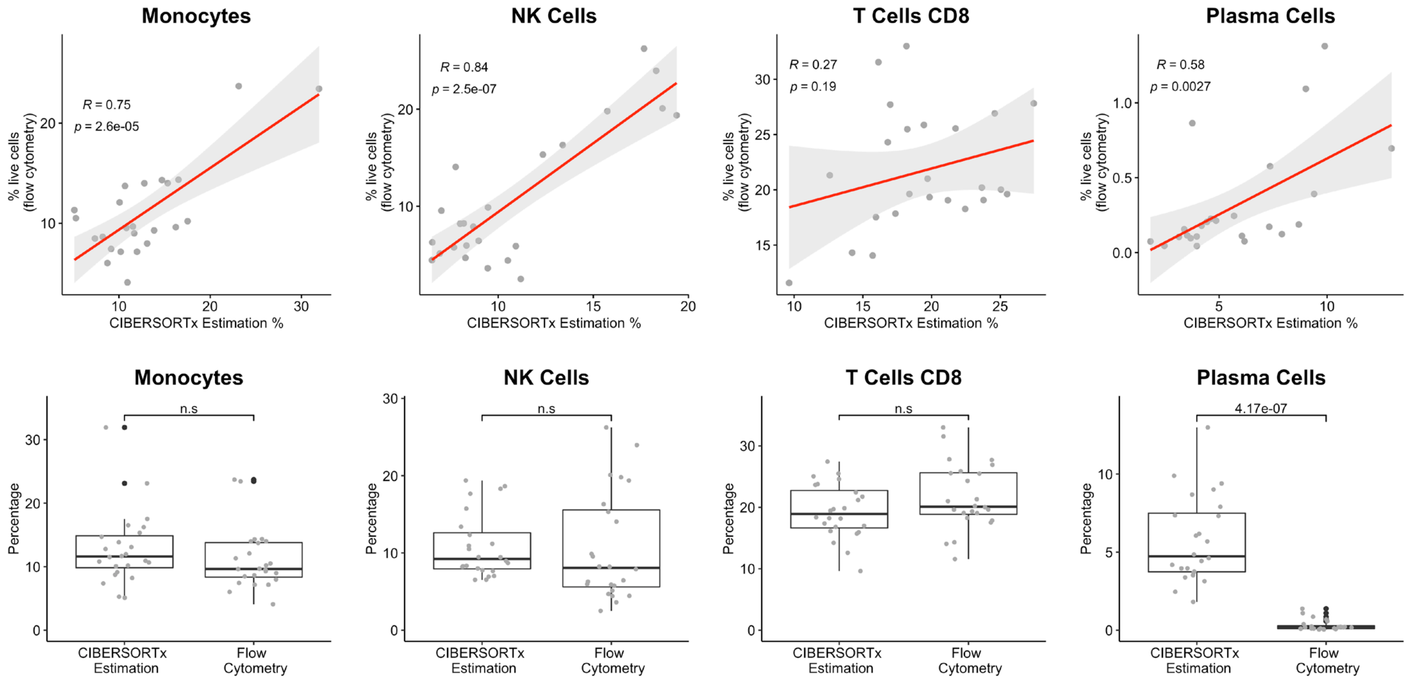

- Pearson's correlations and Wilcoxon 2-sided signed rank tests were applied.
- P-values shown in the boxplots represent Benjamini-Hochberg adjustments.

Expanded Flow Cytometry Methods

Approximately 0.5 x 10^6 to 2 x 10^6 cells per cryopreserved sample were stained for flow cytometry analysis. The titrations of antibodies were previously established by Cytek Biosciences with slight modifications. All staining procedures were performed at room temperature. PBMCs were stained with Live/Dead Blue (Thermofisher) for 15 min, washed with FACS-EDTA buffer and spun down at 1500 rpm for 5min. Samples were resuspended with Brilliant Stain Buffer Plus (BD Biosciences) and stained with a mixture of antibodies for 30 min. Antibodies used in this study include CD45, CD3, CD4, CD8, CD19, CD20, CD56, CD14, CD16, CD27, CD38 and IgD (See the table for details). After incubations, PBMCs were washed with FACS-EDTA buffer and spun down at 1500 rpm for 5min. Samples were fixed with 1% PFA in PBS for 20 min, spun down and resuspended in FACS-EDTA buffer. Samples were acquired using a four-laser Cytek Aurora Spectral Flow Cytometry. Daily quality control (QC) was run using Cytek's SpectroFlo software and SpectroFlo Cytometer QC beads. Single color controls of PBMCs and UltraComp eBeads (ThermoFisher) were used for spectral unmixing and compensation. Unmixed data were analyzed using FlowJo. Gating strategy for T, B and NK lymphocytes (A), monocyte subsets (B), plasmablasts (C) are presented below.

List of Panel Reagents for Flow Cytometry

#	Specificity	Fluorochrome	Clone #	Vendor	Cat #
1	viability	Live/Dead UV Blue	NA	ThermoFisher	L34962
2	CD45	PerCP	H130	ThermoFisher	MHCD4531
3	CD3	BV 510	OKT3	BioLegend	317332
4	CD4	BUV615	SK3	BD	612987
5	CD8	BUV805	SK1	BD	612889
6	CD19	Spark NIR 685	H1B19	BioLegend	302270
7	CD20	Pacific Orange	HI47	ThermoFisher	MHCD2030
8	CD56	BUV737	NCAM19.2	BD	612766
9	CD14	Spark Blue 550	63D3	BioLegend	367148
10	CD16	BUV496	3G8	BD	612944
11	CD27	APC	O323	ThermoFisher	17-0279-42
12	CD38	APC-Fire810	HIT2	BioLegend	Custom
13	IgD	BV 480	1A6-2	BD	566138

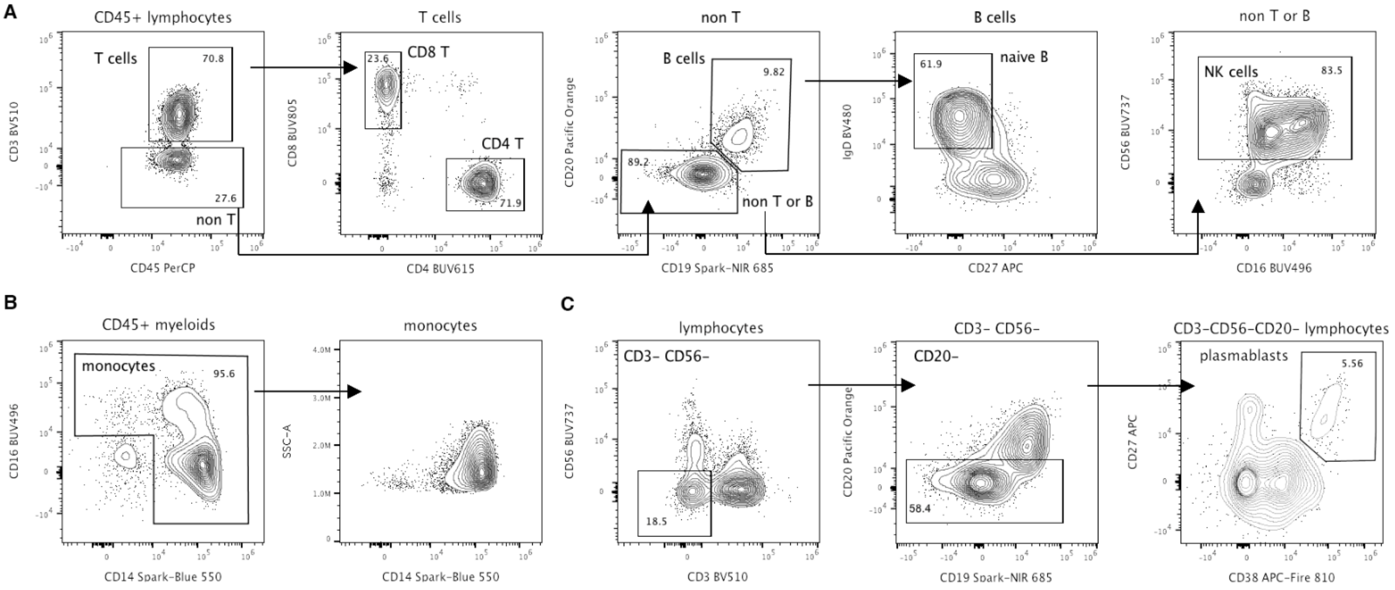

Supplement: Supplementary file 1 — Supplementary Information [file 41467_2021_21289_MOESM1_ESM.pdf]
