# Supplementary material for: Dysregulated transcriptional responses to SARS-CoV-2 in the periphery
Source: Nat Commun. 2021 Feb 17;12:1079. doi: 10.1038/s41467-021-21289-y (PMC7889643; doi:10.1038/s41467-021-21289-y)
Supplement: Supplementary file 2 — Reporting Summary [file 41467_2021_21289_MOESM2_ESM.pdf]

nature research

Corresponding author(s): Micah T. McClain

Last updated by author(s): Dec 15, 2020

Reporting Summary

Nature Research wishes to improve the reproducibility of the work that we publish. This form provides structure for consistency and transparency in reporting. For further information on Nature Research policies, see our Editorial Policies and the Editorial Policy Checklist.

Statistics

For all statistical analyses, confirm that the following items are present in the figure legend, table legend, main text, or Methods section.

n/a	Confirmed	
☐	☒	The exact sample size (*n*) for each experimental group/condition, given as a discrete number and unit of measurement
☐	☒	A statement on whether measurements were taken from distinct samples or whether the same sample was measured repeatedly
☐	☒	The statistical test(s) used AND whether they are one- or two-sided<br>*Only common tests should be described solely by name; describe more complex techniques in the Methods section.*
☐	☒	A description of all covariates tested
☐	☒	A description of any assumptions or corrections, such as tests of normality and adjustment for multiple comparisons
☐	☒	A full description of the statistical parameters including central tendency (e.g. means) or other basic estimates (e.g. regression coefficient) AND variation (e.g. standard deviation) or associated estimates of uncertainty (e.g. confidence intervals)
☐	☒	For null hypothesis testing, the test statistic (e.g. *F*, *t*, *r*) with confidence intervals, effect sizes, degrees of freedom and *P* value noted<br>*Give P values as exact values whenever suitable.*
☒	☐	For Bayesian analysis, information on the choice of priors and Markov chain Monte Carlo settings
☒	☐	For hierarchical and complex designs, identification of the appropriate level for tests and full reporting of outcomes
☒	☐	Estimates of effect sizes (e.g. Cohen's *d*, Pearson's *r*), indicating how they were calculated

Our web collection on statistics for biologists contains articles on many of the points above.

Software and code

Policy information about availability of computer code

Data collection	REDCap 10.0.26, STAR version: 2.7.1a
Data analysis	R version 3.6.1. Packages used contained in README.txt.

For manuscripts utilizing custom algorithms or software that are central to the research but not yet described in published literature, software must be made available to editors and reviewers. We strongly encourage code deposition in a community repository (e.g. GitHub). See the Nature Research guidelines for submitting code & software for further information.

Data

Policy information about availability of data

All manuscripts must include a data availability statement. This statement should provide the following information, where applicable:

- Accession codes, unique identifiers, or web links for publicly available datasets
- A list of figures that have associated raw data
- A description of any restrictions on data availability

Processed data and raw RNA Sequencing data is available at the NCBI Gene Expression Omnibus (accession number: GSE161731)

Field-specific reporting

Life sciences study design

All studies must disclose on these points even when the disclosure is negative.

Sample size	Sample size and experimental design were determined primarily by availability / enrollment of subjects with each desired phenotype (eg, all subjects we had enrolled with COVID-19 were included), the time-sensitive nature of research into COVID-19, and limited by available emergency funding to perform sequencing experiments.
Data exclusions	For signature derivation enrolled subjects were excluded if they had proven co-infections due to multiple pathogens, as this study was not designed or powered to analyze this subset.
Replication	Given the absence of availability of publicly available peripheral blood bulk RNA sequencing datasets at the time of submission, reproducibility was evaluated at the level of statistical modeling through leave-one-out cross validation (LOOCV).
Randomization	Experimental groups were determined by phenotype. Model validations were performed with LOOCV where samples are selected at random from the experimental dataset.
Blinding	Subjects were enrolled with known diagnoses, and the purpose of the study was to directly compare subjects with known infections to other known infection types.

Reporting for specific materials, systems and methods

We require information from authors about some types of materials, experimental systems and methods used in many studies. Here, indicate whether each material, system or method listed is relevant to your study. If you are not sure if a list item applies to your research, read the appropriate section before selecting a response.

Materials & experimental systems

n/a	Involved in the study	
	x	Antibodies
x		Eukaryotic cell lines
x		Palaeontology and archaeology
x		Animals and other organisms
	x	Human research participants
x		Clinical data
x		Dual use research of concern

Methods

n/a	Involved in the study	
x		ChIP-seq
	x	Flow cytometry
x		MRI-based neuroimaging

Antibodies

Antibodies used	Only flow cytometry associated antibodies were used. See relevant information below.
Validation	See below.

Human research participants

Policy information about studies involving human research participants

Population characteristics	Subject demographics and population characteristics are defined in the manuscript in Table s1 and Table s2 in the supplementary material.
Recruitment	Subjects were identified through active surveillance of hospital admissions and outpatient clinic / emergency room visits for suspected COVID-19 or other acute respiratory illness. All subjects admitted to Duke University Medical Center with the relevant diagnoses (COVID-19, influenza, etc) during the timeframe of the study were approached for potential enrollment.
Ethics oversight	The relevant protocols were approved by the Institutional Review Boards of Duke University Medical Center and the Durham Veterans Affairs Medical Center.

Note that full information on the approval of the study protocol must also be provided in the manuscript.

Flow Cytometry

Plots

Confirm that:

- [x] The axis labels state the marker and fluorochrome used (e.g. CD4-FITC).
- [x] The axis scales are clearly visible. Include numbers along axes only for bottom left plot of group (a 'group' is an analysis of identical markers).
- [x] All plots are contour plots with outliers or pseudocolor plots.
- [x] A numerical value for number of cells or percentage (with statistics) is provided.

Methodology

Sample preparation	Frozen PBMCs were thawed and sequentially stained for viability and surface markers, and then fixed in 1% PFA.
Instrument	Cells were analyzed with Cytek Aurora Spectral Flow Cytometry (Cytek Biosciences).
Software	Data were analyzed using FlowJo software Version 10.6.1 (Tree Star).
Cell population abundance	Cell population abundance was shown as either a proportion of live, single PBMCs or a proportion of a parent gate.
Gating strategy	FSC-A and SSC-A were used to select cells and remove debris. Singlets were identified by FSC-A and FSC-H. Viable cells were gated by Live/dead UV blue. Lymphocytes and myeloids were gated by CD45 and SSC-A. Different subsets were further gated for T cells (CD3, CD4 and CD8), B cells (CD3, CD19, CD20, IgD and CD27), NK cells (CD3, CD19, CD20 and CD56), monocytes (CD14, CD16 and SSC-A), plasma cells (CD3, CD19, CD20, CD56, CD27 and CD38).

- [x] Tick this box to confirm that a figure exemplifying the gating strategy is provided in the Supplementary Information.
